# Supplementary material for: Are carbon emissions trading and green financial instruments synergistic? -Comprehensive quantitative research based on content analysis
Source: PLoS One. 2024 Mar 7;19(3):e0298601. doi: 10.1371/journal.pone.0298601 (PMC10919601; doi:10.1371/journal.pone.0298601)
Supplement: S1 Data — (ZIP) [file pone.0298601.s001.zip › Data/地级市Z_F工作报告.zip!/║■─╧/╓Ω╓▐╩╨/└┤╫╘╣1⁄2╓┌║┼í╛┬φ┐╦╩2╛▌═°í┐_2007.docx]

一、2006年工作回顾

　　过去的一年，我们在市委领导下，全面落实科学发展观，紧扣“推进跨越发展，构建和谐株洲”工作主题，大力实施产业兴市、科教先导、城镇带动、文化提升四大战略，全面完成了市十二届人大四次会议提出的各项任务，实现了“十一五”的良好开局。全市实现GDP605.3亿元，增长12.3%，六年翻了一番；完成财政总收入52.5亿元，增长21.3%，四年翻了一番；城镇居民人均可支配收入12455元，增长10.9%，农民人均纯收入4366元，增长10.3%。

　　（一）坚持工业主导，新型工业化进程加快。产业集群逐步壮大。出台并实施了《关于加快产业配套培育产业集群的若干意见》。交通装备制造、有色冶金及深加工、化工、陶瓷、农产品加工和服饰等六大产业集群，实现增加值158亿元，增长16.2%，占工业增加值的比重提高到58%。株冶集团跻身全省七大年销售收入过100亿元企业，时代电气成为我市第一家、全省第二家在港上市公司。全部工业完成增加值270.6亿元，增长14.6%，占GDP比重提高到44.7%，位居全省第一；规模工业实现净利润24.3亿元，增长81.2%，工业经济效益综合指数达到153.8，刷新历史纪录。技改力度持续加大。实施项目767个，完成投资76.8亿元，增长42.7%。株洲旗滨玻璃集团日产700吨优质浮法玻璃工程、株化20万吨PVC一期扩改工程等顺利投产。株硬集团高性能硬质合金工具工程、株冶集团锌冶炼节能降耗技术改造等进展顺利。编制并实施了工业循环经济发展规划，全面完成了主要污染物年度减排任务。园区实力进一步增强。推行园区目标考核，继续拼盘投入4000万元，支持整体招商和技术创新，加大基础设施和产业项目建设力度。各园区共完成固定资产投资52.4亿元，增长32.2%，实现技工贸总收入292.7亿元，增长11.4%。

　　（二）坚持城乡统筹，社会主义新农村建设全面铺开。农业产业化水平提高。粮食播种面积388.5万亩，粮食总产量187.2万吨，是历史上第二个高产年。出栏生猪466.2万头，增长5.5%。引进5000万元以上项目3个。实现农产品加工产值78亿元。“十百千”工程成效明显。投入300多万元，全面完成了9个县市区的村庄布点总体规划和116个村庄整治建设规划。投入8000多万元，帮扶105个示范村改善基础设施，发展特色种养。以“三清四改”为重点，全面开展了千村整治。农业综合生产能力不断增强。水利建设投入3.47亿元，连续五年夺得“芙蓉杯”。洮水水库成功截流。生态建设完成工程造林5.6万亩，其中退耕还林4.2万亩，森林覆盖率提高到59.21%。沼气用户突破5万户。农业综合开发投入6000多万元，改造中低产田6.4万亩。改造农村公路1700多公里，建设客运站22个。县域经济实力增强。五县市实现GDP320亿元、财政总收入17.8亿元，分别占全市的52.9%、33.9%。

　　（三）坚持城镇带动，基础设施更加完善。城市建设跃上新台阶。第六次城市总体规划（修编）经国务院批准实施。中环大道建成通车，为建设特大城市拉开了骨架，拓展了空间。湘江四桥基本建成。建宁大道基本完成征地拆迁，沿江防洪景观道路、市府路涵洞、新华桥扩改进展顺利，人民北路、杨栗线、黄河北路跨西环线桥梁主体、老文化路基本完工，枫溪大道、新华东路延伸段竣工通车。白石港流域治理、枫溪港堤防建设稳步推进，天鹅湖、建宁港治理一期工程顺利完成。小城镇建设迈出新步伐。提质扩容速度加快，共完成投资9.7亿元，增长11.1%，全市城镇化水平提高到46%。交通能源建设掀起新高潮。共完成交通建设投资20亿元。醴潭高速路基工程全面完成，长株、衡炎高速开工建设，武广客运专线完成年度计划。网朱公路、S313醴陵至株洲县、S321炎陵至睦村、S320茶陵至界化垄等4条干线公路进展顺利。电网建设完成投资3.5亿元，沔渡、黄兰等输变电工程投产送电。全市仅存的1573户“油灯户”户户通电。全年完成固定资产投资203.2亿元，增长26.5%，实现两天投资1.1亿元，四年翻了一番。

　　（四）坚持与时俱进，改革开放实现突破。各项改革不断深化。列入扫尾计划的17家市属国有工业企业改革进展顺利。在株省属国有企业的社会职能分离步伐加快。以乡镇机构、义务教育和财政管理体制改革为重点的农村综合改革全面铺开。公共财政体系更加完善。城市管理相对集中了行政处罚权。“同城合一”的招投标市场业已形成。事业单位改革拉开序幕。对外开放继续扩大。新批外商投资企业67家，实际到位外资1.9亿美元，增长20.2%，利用市外境内资金81亿元，增长33.1%。完成进出口总额9.67亿美元，增长40.5%，其中出口8.13亿美元，增长49.4%。新增境外投资企业5家。城乡市场更趋活跃。实现社会消费品零售总额208.5亿元，增长15.1%。金融机构年末各项存款余额552.4亿元，新增79.9亿元，贷款余额254.5亿元，新增19.5亿元。运输邮电业增加值30.8亿元，增长12.4%。房地产业增加值18亿元，增长8.3%。中国优秀旅游城市创建成功。同时，非公经济蓬勃发展，实现增加值318亿元，增长12.3%。

　　（五）坚持科教先导，社会事业协调发展。科技创新能力不断增强。支持企业提高自主创新能力，共有50个项目申报为国家、省级科技项目，3个项目获国家科技进步二等奖。新增高新技术企业22家，实现高新技术产品产值228.9亿元，增长34.7%。株洲被列为国家专利（轨道交通）产业化试点基地。各级各类教育健康发展。投入1.5亿元，顺利完成了城区基础教育管理体制调整，城区义务教育全部下放到区。筹资2900多万元，对10万余名贫困学生“两免一补”。铁道职院成为国家示范性高职院校。引资1.5亿元，发展民办教育。公共卫生体系更加完善。投资2200多万元，新改扩建乡镇卫生院44所。炎陵、茶陵、醴陵三县（市）农村合作医疗参合率89%，有5万多农民享受医疗补助3700多万元。建成社区卫生服务中心18个、卫生服务站36个，社区卫生服务机构覆盖面达90%。其他事业繁荣有序。体育中心投入使用，承办省十运会和第七届残运会取得圆满成功，体现了株洲实力，彰显了城市风采。广电集团成功组建，广电中心顺利开工，广播电视“村村通”全面推进。“扫黄打非”工作荣获全国先进。“炎帝陵祭祀”被列入国家非物质文化遗产名录，文化园世纪广场被评为“全国特色文化广场”。

　　（六）坚持以人为本，社会和谐程度得到提高。劳动和社会保障体系日趋健全。新一轮就业政策全面落实，共有2.5万人享受了税收优惠或社会保险补贴，全市所有“零就业家庭”每户至少1人实现了再就业。治理拖欠农民工工资工作获得全国先进。社会保险和住房公积金覆盖面不断扩大。醴陵、茶陵农村低保试点顺利启动，全市共发放农村特困户救助金204.1万元。投入扶贫开发资金2018万元，帮助6000人脱贫。经济适用房货币补贴在全省率先启动。人口资源环境工作得到加强。推行农村计生家庭奖励、扶助政策，共有4918户家庭受益，计划生育率95.72%。严格执行国土资源政策，耕地占补实现平衡。全力推进城区环境污染综合治理和“蓝天行动”，市区空气良好天数308天，比上年增加10天，市区饮用水源水质合格率提高到98.1%。社会大局基本稳定。“大信访”格局初步构建。“五五”普法全面铺开。社会治安综合治理工作再获全省先进。生产、消防、交通安全专项整治不断深化，安全生产事故起数下降16.7%，杜绝了特大火灾事故和特大交通事故。同时，投入3.49亿元，圆满完成了年初确定的10件实事。

　　（七）坚持依法行政，政府职能进一步转变。“企业服务年”活动成效明显。企业提出的问题，100%得到答复，80%得到解决。设立中小企业专项资金，加大政银企合作力度，提供特种政策补助资金8000万元。支持企业获得中国名牌产品1个、中国驰名商标1件。依法行政水平和工作效能不断提高。政企、政事、政资、政府与中介机构“四分开”进程加快。承办人大代表建议242件、政协委员提案227件，办结率和见面率均为100%，满意率99.4%。开辟“绿色通道”，推进联合审批，缩短了办事时限。建成市政府网络数据中心，开通了党政办公业务资源网。社会管理和公共服务力度加大。对国有资产、食品、药品和社会安全等方面的监管得到加强。城市和社区管理日益规范，深入开展了城区环境综合整治。严厉打击了偷税漏税、商业欺诈、欺行霸市等不法行为。同时，积极开展国防教育和“双拥”活动，人防、民兵预备役工作得到加强。

　　二、2007年工作总体要求

　　当前，我们面临难得的发展机遇和广阔的发展空间。新的一年，我们将紧扣本届政府任期目标和“十一五”规划，突出“推进跨越发展，构建和谐株洲”工作主题，全面实施“四大战略”，着力加强基础产业、基础设施和基础工作，着力推进新型工业化，着力加强节能降耗和生态环境保护，着力推进自主创新和项目建设，着力促进社会和谐，努力实现又好又快发展。

　　全年经济社会发展的主要预期目标是：GDP增长12.5%，财政总收入增长13%，全社会固定资产投资增长20%，社会消费品零售总额增长14.5%，城镇居民人均可支配收入增长9%，农民人均纯收入增长6%，新增城镇就业4.7万人，城镇登记失业率控制在4.5%以内，万元GDP能耗下降4%，主要污染物排放总量减少4%，人口自然增长率控制在7.4‰以内，城市化水平提高到47%。各项事业都有新的进步。

　　2007年，我们将按照市委的统一部署，深入开展“项目建设年”活动。项目是产业的载体、财税的源泉、后劲的依托。抓项目就是抓发展，抓项目就是促和谐。我们务必以“项目建设年”为契机，精心策划谋项目，支持企业上项目，千方百计争项目，优质服务助项目，以项目建设的大推进，实现产业的大发展、基础设施的大改善、社会事业的大进步。

　　三、以新型工业化为动力，加速推进跨越发展

　　新型工业化是加快发展的第一推动力。推进新型工业化，既离不开产业的支撑，又离不开城镇的带动。新的一年，我们将继续坚持非均衡发展理念，按照布局集中、产业集群、资源集约的要求，大力实施产业兴市和城镇带动战略，为加快推进新型工业化锻造骨架，夯实平台，带动经济又好又快发展。

　　——加快推进新型工业化，提高发展水平

　　突出项目带动，壮大优势产业。支持中小航空发动机基地、风力发电装备制造产业化、大功率交流传动电力机车产业化、高速铁路道岔、株冶集团循环经济建设工程等项目建设，促进电厂B厂等重大项目早日开工。同时，认真贯彻落实《中小企业促进法》，支持发展为优势产业提供配套服务的中小企业。加快园区建设，培育产业集群。全面落实各项优惠政策，拼盘投入5000万元，加快株洲高新区、湖南欧洲工业园、清水塘循环经济区和醴陵陶瓷科技工业园建设，继续支持每个县市区建好一个特色工业园区。扩大园区产业链招商，发展配套产业，延伸产业链条，培植产业集群。繁荣现代服务业，支撑产业发展。充分发挥现代服务业的支撑和推动作用，规划、建设、完善一批生产资料、生活用品、仓储配送等物流设施，加快发展生产性服务业。全面实施市商业网点规划，推进“万村千乡”市场工程，改造提升商贸服务业。巩固“创优”成果，加快景区开发，打造以炎帝陵为代表的人文旅游和以醴陵陶瓷为代表的工业旅游品牌，壮大旅游产业。规范发展房地产、中介、物业管理等专业服务业。

　　——建设社会主义新农村，拓展发展空间

　　大力发展现代农业，努力促进村民富。全面落实支农惠农政策，切实加大“三农”投入。支持壮大一批产业化龙头企业，突出抓好106国道沿线优势农业产业带建设，发展城郊休闲农业，带动提升竹木、肉食等优势产业，用产业化理念经营农业；继续大力推广先进适用技术和优良品种，不断完善农村社会化服务体系，提高农产品质量和农业比较效益，用先进科学技术提升农业；认真实施《农产品质量安全法》，引导建设一批无公害、绿色和有机食品生产基地，用标准化体系建设农业。发展农村二、三产业和劳务经济，推动农村富余劳动力转移就业。继续实施“十百千”工程，努力促进村庄好。严格实施已编制的示范村村庄规划，完成300个村庄规划编制。加大对105个示范村和10个重点村的帮扶力度，完成1000个村的村容村貌整治。加强农村基础设施和公共事业建设，不断改善农民生产生活条件。着力培育文明乡风，努力促进村风正。落实村民自治，扩大基层民主。加强民主法制教育，大力倡导村规民约。加强农民适用技术和职业技能培训，抓好农村精神文明创建和道德评议活动，努力培养有知识、讲道德、懂技术、会经营的新型农民。

　　——不断完善基础设施，夯实发展平台

　　城区方面，认真实施第六次城市总体规划（修编）。加快湘江城区段防洪景观道路、建宁大道、枫溪大道延伸段、黄河北路、铁西南路、芦淞路、泰山路、株醴路延伸段、东环北路等主次干道的建设或改造，完善中环大道、人民北路的配套设施，湘江四桥竣工通车。做好炎帝大道的前期工作。完成建宁港桥、红港路1号和3号铁桥改造。推进河西污水处理厂建设。继续加大城区排水管网改造力度，提质改造小街小巷。农村方面，加强农田水利基本建设，重点搞好湘渌堤防工程，完成62座中小型水库的病险治理和灌区的节水改造工程，改造中低产田6万亩。工程造林6.6万亩，全市森林覆盖率提高到59.58%。继续加强小城镇建设。交通能源方面，支持醴潭、衡炎、长株高速和武广客运专线株洲段建设。基本完成网朱、S313醴陵至株洲县、S321炎陵至睦村、S320茶陵至界化垄等干线公路的路基工程。做好衡炎高速攸县连接线、S322资兴至牛岗排、醴潭高速株洲东互通至黄塘公路的前期工作。加快洮水水库建设，争取铜塘湾港区一期工程早日动工。继续推进城乡电网改造，加快发展小水电、沼气等农村生态能源。

　　——全面推进改革开放，增强发展后劲

　　继续深化各项改革。基本完成市属国有企业改革扫尾任务，配合做好在株中央、省属国有企业社会职能剥离工作。完善国有资产监督管理体系。继续加大公共财政体制、投融资体制以及行政审批制度改革力度，深入推进农村综合改革、公用事业改革和要素市场改革。大力发展开放型经济。创新招商方式，完善招商机制，重点引进关联度大、聚集度高、带动力强、对地方财政和就业贡献大的项目。力争全年利用外资2.2亿美元，利用内资90亿元。千方百计扩大出口，鼓励有条件的企业开展对外投资、跨国经营、劳务输出。认真落实有关政策措施，加大信贷和财税等方面的扶持力度，重点支持创新能力强、就业容量大的非公企业，做大做强非公经济。

　　四、以人本理念为指导，加快构建和谐株洲

　　建设社会主义和谐社会是新的伟大长征。构建和谐株洲，既需要经济的快速健康发展，又需要事业的全面进步。新的一年，我们将继续坚持以人为本的理念，顺应人民群众求富裕、求公正、求安定的愿望，大力实施科教先导和文化提升战略，有效协调各方利益，改善各方关系，努力形成全市人民各尽所能、各得其所而又和谐相处的良好局面。

　　——强化载体建设，发展社会事业

　　大力推进自主创新。坚持把自主创新作为推进新型工业化的战略支撑，进一步完善以企业为主体、市场为导向、产学研相结合的自主创新体系，建成首批市级工程技术研究中心5家，培育制造业信息化示范企业10家，启动市级创新型企业试点工作，加快建设创新型城市。大力加强科技创新服务平台和网上技术市场建设，促进优秀科技成果向现实生产力转化。加快发展教育事业。加大基础教育投入，做好“两基”迎国检工作。加快中小学布局调整和学校改造，推进城乡区域教育均衡发展。全面落实农村义务教育经费保障新机制，对贫困家庭学生继续实施“两免一补”。深化教育体制改革，促进民办教育健康发展。加快发展职业教育。支持在株高校做大做强。努力提高健康保障水平。进一步完善疾病预防控制体系和公共卫生应急反应机制。规范发展民营医疗机构，加强医疗市场监管。加快建设河西中心医院，积极筹建市二医院、人民医院新门诊综合大楼。建成24个社区卫生服务中心和32个服务站。加强群众性体育设施建设，办好第三届大众运动会。不断丰富文化生活。开工建设株洲影剧院，开展首届"读书月"活动。整治文化市场，实施文化环保工程。办好丁亥年重阳炎帝陵祭祖大典。加快建设市广播电视中心，推进城区有线电视数字化整体转换。建成启用新档案馆。全面落实民族宗教政策。做好第二轮修志工作。

　　同时，加强国防动员、人防和民兵预备役建设，继续做好双拥优抚安置工作，巩固军政军民团结。

　　——关心群众生活，维护民生民利

　　千方百计扩大就业。进一步健全劳动者自主就业、市场调节就业和政府促进就业机制。落实各项优惠政策和就业援助制度，帮助有就业愿望的新增“零就业家庭”实现每户至少1人就业。加强劳动力职业技能培训，完善人力资源市场。提高社会保障水平。深化养老保险制度改革，逐步做实个人账户，提高基金支付能力。着力推进社会保险向自由职业、灵活就业人员的扩面工作。积极探索农村养老保险和失地农民社会保障办法。加强扶贫济困助残工作。认真实施产业扶贫。巩固城乡最低生活保障制度。推行城乡医疗救助制度，着力缓解因病致贫、因病返贫问题。重视残疾人工作，支持发展慈善事业。做好法律援助工作，帮助困难群众维护合法权益。

　　——落实基本国策，坚持可持续发展

　　加强人口与计划生育工作。稳定低生育水平，提高人口素质，改善人口结构。强化流动人口管理，严厉打击“两非”违法行为。认真实施妇女儿童发展规划，完善老年服务体系。建设青山秀水的生态环境。坚持治理与保护并重，注重节约土地、能源、水资源，保持耕地占补平衡，集约开发利用矿产资源。大力发展循环经济，推广清洁生产，抓好重点领域、重点行业和重点企业的节能降耗。切实加大保护湘江力度，综合整治城市环境，从源头上控制污染，全年空气良好天数力争290天以上，市区饮用水源水质达标率稳定在96%以上。

　　——坚持务实为民，办好惠民十件实事

　　着眼于群众切身利益，继续办好惠民十件实事：1、健全农村交通、广播电视和流通网络，建设或改造农村公路1200公里；推进农村广播电视“户户通”工程，实现4万户农户无线数字电视入户；建设210家标准化乡村农家店。2、进一步改善农村办学条件，落实农村义务教育经费保障新机制，全部免除农村义务教育阶段学生学杂费；筹资1300万元，救助8万名以上家庭贫困学生。3、完善农村卫生服务体系，新改扩建乡镇卫生院34所；在五县（市）全面推进新型农村合作医疗制度，参合率在80%以上，启动天元区试点工作。4、继续实施计划生育奖励扶助制度，农村计划生育家庭奖励扶助金、独生子女死亡伤残家庭扶助金发放率和符合条件的再生育夫妇施行出生缺陷干预到位率均达到100%。5、做好城乡就业和社会保障工作，新增城镇就业4.7万人,下岗失业人员再就业培训1.5万人，再就业2.3万人；农村实用技术培训10万人次，新增转移就业5万人；企业养老、失业、医疗和工伤保险参保人数分别新增3.55万人、1.55万人、2.5万人和4.5万人。6、提高城乡社会救助水平，城市“低保”标准提高到190元，资金拨付100%到位；全面实行农村最低生活保障制度，农村“低保”标准由15元提高到30元，新改扩建乡镇敬老院10所。7、大力整治城乡环境，城市污水处理率和生活垃圾无害化处理率分别达到55%和100%；完成老霞湾港和铜塘港的污染治理，提质改造南湖塘路、建湘路等11条城市小街小巷；新建农村沼气池6000口；解决农村1万人饮水安全问题。8、加强公共服务设施建设，建成食品药品安全检测中心；启动株洲艺术中心建设；改造河东体育场馆，争取建设国家级全民健身基地。9、加大法律援助工作力度，为经济困难群众、弱势群体提供1000例以上法律援助。10、做好园林城市创建和迎检工作，新增城区绿地面积94公顷，建成区绿地率、绿化覆盖率分别达到36%、40%，人均公共绿地面积达到9.5平方米，力争通过国家园林城市检查验收。

　　五、以职能转变为根本，加强政府自身建设

　　转变职能是政府自身建设的永恒课题。加快转变政府职能，既要加强社会管理，又要优化公共服务。新的一年，我们将继续坚持“为所当为”的施政理念，瞄准高效廉洁、勤政为民的目标，把工作重点转移到为市场主体服务和创造良好发展环境上来，努力建设法治政府、责任政府、诚信政府和服务型政府。

　　努力提高执政水平。健全公众参与、专家论证和政府决策相结合的决策机制，力求决策的科学性。推进政府信息公开和电子政务建设，增强政务的透明度。继续办好政务中心，加大重点项目效能监察和优化发展环境力度，提高决策的执行力。继续深入实施《行政许可法》，进一步规范行政行为。加强管理和培训，进一步改进工作作风，建设一支高素质的公务员队伍。切实加强社会管理与建设。认真落实社会治安综治措施，加强公安基层基础建设，依法打击各类违法犯罪行为。深入开展“强化城管，提升文明”行动，进一步加强城市和社区管理。健全安全生产工作机制，完善监管网络，突出抓好重点行业、重点领域的专项治理，做好交通和消防安全工作。强化食品药品质量安全管理，让广大群众吃上放心食品，用上放心药品。加强应急管理，提高应对群体性事件的预防和处置能力。畅通民意诉求渠道，有效协调各方关系。继续开展“五五”普法，高度重视信访和人民调解工作。认真做好第二次农业经济普查。自觉接受人民监督。主动接受市人大及其常委会的监督，认真听取人民政协、各民主党派、工商联、无党派人士和各人民团体的意见。自觉接受新闻舆论监督和社会公众监督。支持监察、审计部门依法履行监督职责。进一步加强与法院、检察院等国家机关的联系。
